# A qualitative exploration of the health needs and goals of urban women to inform the tailoring and adaptation of Strong Hearts Healthy Communities: a community-based cardiovascular disease prevention intervention

**DOI:** 10.1186/s12889-024-17818-1

**Published:** 2024-01-25

**Authors:** Phrashiah Githinji, Alexandra L. MacMillan Uribe, Rebecca A. Seguin-Fowler, Priscilla Ayine, Anita On, Deyaun L. Villarreal, Andrew McNeely, Jacob Szeszulski, Chad D. Rethorst

**Affiliations:** Institute for Advancing Health Through Agriculture, Texas A&M University, College Station, Dallas, TX USA

**Keywords:** Cardiovascular disease, Intervention adaptation, Health promotion, Urban, Women, Health behavior, Nutrition, Physical activity, Stress, Minority health

## Abstract

**Background:**

In the United States, cardiovascular diseases (CVD) are the leading cause of death and disability in women. CVD-modifiable risk factors, including poor diet quality and inadequate physical activity, can be addressed through evidence-based interventions (EBIs). Strong Hearts Healthy Communities (SHHC) is an EBI that has demonstrated effectiveness in reducing CVD risk and improving health outcomes among rural white women. The aims of this study were to understand the general health, diet, and physical activity-related needs and goals of women living in an urban community, to inform the tailoring and adaptation of the SHHC EBI to an urban setting and more diverse population.

**Methods:**

Focus groups (FGs) were conducted with African American/Black and Hispanic/Latinx women in the Dallas metropolitan area who had a BMI ≥ 25 kg/m^2^ and engaged in ≤ 150 min per week of moderate physical activity. The data were coded using a team-based, deductive, and thematic analysis approach, that included multiple coders and in-depth discussions.

**Results:**

Four FGs with a total of 18 participants (79% Black and 21% Latinx) were conducted, and three themes were developed: (1) participants had adequate knowledge and positive attitudes towards healthy living but faced many barriers to practicing healthy behaviors; (2) culturally-based beliefs and community practices exerted a strong influence on behaviors related to food and stress, revealing barriers to healthy eating and generational differences in stress and stress management; (3) participants desired a more individualized approach to nutrition and physical activity interventions that included familiar and enjoyable activities and social support centered around shared health goals.

**Conclusions:**

The SHHC intervention and similar health programs for Black/African American and Hispanic/Latinx women in urban settings should emphasize individualized nutrition and practical skills for healthy eating with accessible, familiar, and enjoyable exercises. Additionally, stress management strategies should be culturally and generationally sensitive and social support, whether through family, friends, or other program participants, should be based on shared health goals.

**Supplementary Information:**

The online version contains supplementary material available at 10.1186/s12889-024-17818-1.

## Background

Cardiovascular diseases (CVD) are the leading cause of death for women in the United States [1]. Approximately 58.8% of non-Hispanic Black females and 42.7% of Hispanic females aged 20 years or older have CVD [1]. One reason Black/African American and Hispanic/Latinx women are disproportionately affected by CVD is a higher prevalence of lifestyle-related risk factors, such as poor diet quality and physical inactivity [2, 3]. Addressing these lifestyle risk factors through community-level interventions can reduce CVD risk, particularly for underserved and minority populations [4].

One way to address CVD-related risk factors is to deliver evidence-based interventions (EBIs) that improve behavioral and health outcomes. Whether targeting whole populations or specific groups, health interventions are complex because they operate through active contextual interactions with significant variations across different populations and settings [5]. Strong Hearts Healthy Communities (SHHC) is a 24-week multilevel EBI that has been demonstrated to be effective in reducing CVD-related health behaviors and outcomes among rural women living in medically underserved communities [6]. SHHC focused on providing strength training, aerobic exercise, skill-based nutrition education and civic engagement components related to healthy food and physical activity environments. In intervention versus control participants, significant improvements were observed in body weight, body mass index (BMI), C-reactive protein and Life’s Simple 7 CVD risk score [6].

Adapting EBIs that have proven effective in improving health behaviors and outcomes can help in the prevention or management of chronic diseases and save resources associated with developing new interventions for each context [7]. Furthermore, research evidence has demonstrated the benefits of tailored EBIs compared to EBIs that were developed in a different setting or context [8]. For instance, interventions tailored to a specific population’s culture, goals, needs, values, and beliefs are more likely to be accepted and utilized than those created without considering a population’s needs [9, 10]. Additionally, understanding the needs of the population of interest, can help to better inform the design and implementation of health interventions [10]. However, while the tailoring and adapting EBIs may involve assessing the population of interests’ socio-environmental context, goals, needs, values, beliefs, history, traditions, or language and using this information to tailor the intervention based on that knowledge, the decisions of what, when and how to adapt an EBI are not straightforward [11, 12].

Previous research has not fully explored the perceptions, values and experiences that shape the health goals and needs of Black/African American and Hispanic/Latinx urban women. Therefore, to access the necessary information to tailor and adapt the SHHC program to an urban setting and a more diverse population, we conducted qualitative focus groups (FGs) to gain a current and in-depth understanding of the general health, diet, physical activity, and stress-related needs and goals of Black/African American and Hispanic/Latinx urban women.

## Methods

### Study design, setting, and sample

We conducted a qualitative study utilizing focus group (FG) discussions with participants from Dallas, Texas, USA, an urban city with a diverse population, from June-August 2022. To guide the FG discussions, a semi-structured guide was developed based on a review of previous literature on lifestyle behaviors that improve cardiovascular health, emphasizing health goals, healthy eating, stress management, and food and physical activity (see supplementary file). The FG guide also assessed which components of the current SHHC EBI were most relevant, acceptable, and preferred by the participants. The Texas A&M University Institutional Review Board approved all the materials and procedures (IRB2022-0671 M).

### Participants, recruitment and data collection

Individuals who met the following inclusion criteria were eligible to participate: [1] identified as a woman; [2] were ≥ 18 years old; [3] identified as Black/African American and/or Hispanic/Latinx; [4] had a BMI ≥ 25 kg/m^2^ categorized as overweight or obese; [5] and were engaged in less than 150 min of moderate physical activity per week. Participants were recruited virtually by emailing a Texas A&M AgriLife Research repository of community members interested in research opportunities and through in-person recruitment at community events and partnering with community centers. Two research team members trained in qualitative methods conducted the FGs at the Texas A&M AgriLife Research Center in Dallas and at the West Dallas Multipurpose Center. One researcher was the lead moderator who facilitated discussions (DLV), while the other served as a co-moderator, ensuring that a detailed account of the discussions was captured (CR). Participants completed a questionnaire prior to the FGs in which they provided demographic information and were asked about their current health goals and interest in particular aspects of the SHHC program. Additionally, the questionnaire prompted participants to self-assess their overall health status, with options ranging from excellent to poor. The FGs were audio recorded and lasted approximately 60 min each, following an open-ended FG discussion guide (see supplementary file).

### Data analysis

All focus group discussions (FGs) underwent professional transcription by Ubiqus Reporting, Inc. The transcribed data were then imported into NVivo (QSR Version 12) software to facilitate the subsequent analysis process. Guided by the research objectives, the authors (PG and ALMU) initiated the data analysis process by creating an a-priori codebook based on the questions used in the focus group guide. Employing a content analysis approach along with a team-based coding strategy [13], the research team employed a four-member group to individually analyze one FG transcript each. These analyses involved line-by-line coding, with each team member generating summaries for each a-priori code based on the excerpts assigned to that specific code. Collaborative discussions were held among the team members to address emerging concepts, reach consensus, and incorporate changes to the codebook that reflected consensus-building and the emergence of new concepts. This iterative process ensured a thorough exploration of concepts and ensured that the identified codes were sensitive to the subtleties of the participants’ responses. This rigorous approach led to the achievement of code saturation, denoting that no new codes were being generated from the data.

Subsequent to the initial round of discussions, the transcripts underwent a second round of coding. This time, each transcript was analyzed by a different research team member, using the updated codebook. A subsequent discussion of the newly generated code summaries followed. This iterative approach was for the purpose of enhancing the trustworthiness and credibility of the analysis process through expert triangulation of the identified codes and themes, whereby multiple perspectives and interpretations are incorporated to reduce individual research bias and contribute to the depth and accuracy of the analysis [13]. Post-coding discussions involved the convening of the research team to identify the most prevalent concepts, extract high-level themes and sub-themes from the code summaries, and ensure the interrelationships were accurately represented. Finally, members of the research team (ALMU, CDR, PG, PA, AO) convened to refine and approve the final themes, solidifying the alignment of the themes with the depth and complexity of the data.

## Results

### Participant characteristics

Four FGs with 4–5 participants each were conducted. Participants (*n* = 18) were African American/Black (79%) and Hispanic/Latinx (21%) women, aged 18–65 years. Most participants reported that their health status was “good” or better (Table 1).

**Table 1:** Demographic and health characteristics of participants (*n* = 18)

	n (%)
**Age range**	
18–34 years 35–49 years 50–65 years	3 (16.67%) 8 (44.44%) 7 (38.89%)
**Race** Black/African American Hispanic/Latino	
	15 (83.33%) 3 (16.67%)
**Ethnicity**	
Hispanic or Latino	3 (16.67%)
Not Hispanic or Latino	14 (77.78%)
Not specified	1 (5.55%)
**Perceived health status**	
Excellent Very good Good Fair Poor	4 (22.2%) 6 (33.33%) 7 (38.89%) 0 (0%) 1 (5.46%)

The following themes on cardiovascular health related lifestyle behaviors were identified: (1) participants have adequate knowledge and positive attitudes toward a healthier lifestyle but face many barriers to practicing healthy behaviors; (2) culturally based beliefs and community practices exert a strong influence on behaviors related to food and stress.; (3) participants desire a more individualized approach to nutrition and physical activity interventions.

### Theme 1: participants have adequate knowledge and positive attitudes toward a healthier lifestyle but face barriers to practicing healthy behaviors

Almost all participants had a general awareness that engaging in healthy eating behaviors and physical activity was beneficial for their health. Participants expressed awareness that they needed to eat healthfully by reducing their intake of processed and sugar-sweetened foods such as sweets and desserts and reducing overall caloric intake. Many also stated that physical activity was beneficial in increasing their energy and getting stronger. Furthermore, most participants asserted that maintaining healthy eating and physical activity behaviors could help manage or prevent chronic diseases like hypertension, reduce chronic inflammation and pain, and reduce prescription medication use. Additionally, most participants had a positive attitude towards behavior change, and they expressed a willingness to make healthy lifestyle changes, lsuch as being more active and getting more sleep, as highlighted in the following excerpts:

*I do walk more, and when I walk, it helps decrease the pain, besides the medication (Participant #2, FG #3).*

*I see a lot of research concerning getting better sleep when it comes to Alzheimer’s and Dementia. I’m a night owl and an insomniac. My brain is telling me you need to do better to live a longer and healthier life. (Participant #3, FG #3).*

*I take two blood pressure medications…my doctor said I can eventually get off if I lose 20 pounds. So, losing weight should probably be [a health goal] too. (Participant #1, FG #1).*

*I know that as you age, if your muscle tone is better then, your balance is better. I’ve seen elderly relatives fall and it’s really bad when you fall and try to mend your bones…so, I want to get on a weightlifting regimen just to strengthen my own body. (Participant #4, FG #1).*

Despite having knowledge and positive attitudes toward a healthier lifestyle, participants expressed having challenges in initiating and maintaining healthy lifestyle behaviors. Barriers to healthy eating included cravings for unhealthy foods, financial constraints, and time. For example, one participant shared that they often found themselves in a cycle of eating unhealthy foods because of financial constraints: “*It’s hard to find healthy food that’s not expensive, and then that affects my healthy eating. The price in McDonalds is a lot cheaper than getting a salad from somewhere.*” Barriers to physical activity included weather changes (e.g., hot summers in Texas) and, similar to healthy eating, financial constraints and limited time. Regarding weather-related barriers, one participant shared, “*I want to be more physically active, and we go to the park in the evenings. But it is so hot here, so it’s hard to get outside and to be active, but I want to get more active.*” Furthermore, major life events– such as attending graduate school, having stressful family events, or physical injuries– were significant obstacles for many participants, as they hindered healthy eating, physical activity, and mental health-related activities. For example, participants shared:

*I used to do the 5k’s and 10k’s, and then I fell and hurt my knee, and since hurting my knee and having surgery, I have not been able to get back to my previous level of exercise. (Participant #4, FG #3).*

*Right now, I’m in grad school and I ended up going through a lot of mental health stuff when COVID happened. I started trying to balance family life, working full time, being a new grandmother and everything else. It’s kind of balancing out, but not as much. I feel that stress is what made everything worse with the whole weight gain as I haven’t really been eating right, sleeping right, or even exercising, which I really love to do. (Participant #3, FG #1).*

### Theme 2: culturally-based beliefs and community practices exert a strong influence on behaviors related to food and stress

#### Sub-theme 1: specific cultural beliefs and practices around food present barriers to healthy eating

There was a strong link between culture and food among participants. Participants often shared that food was viewed as an expression of love and a sense of community and belonging within their culture. Additionally, many shared that food served as an anchor to their culture. For most participants, social interactions with family and friends were often centered around food as this was viewed as an expression of love; food was usually provided to show care, hospitality, and affection. Despite this cultural connection between food, love and community, most participants shared that these foods were often unhealthy, and characterized by high fat or added sugar, and large serving sizes were often encouraged. For example, some participants shared the following:

*I grew up with my grandparents and everything revolved around food. I remember I was probably around seven years old, if not younger, and cooking was my grandmother’s love language…I had to grow out of cooking the way she cooked, because everything was deep fried in flour and butter, and other unhealthy things. I realized when I was older those things might taste good, but they aren’t good for you. (Participant #1, FG #4).*

*I used to joke that my mother-in-law has a side of veggies with her salt. She heavily seasons everything and every gathering there’s tons of food and its always food that’s not the healthiest. She says that that’s the way you show love when my kids and grandkids are coming over. We have to make what everyone loves, and everyone loves calorie laden foods. So, if I bring a salad, I’m going to get some looks there. (Participant #1, FG #2).*

A few participants mentioned that healthy eating was rarely discussed during family gatherings despite many family members having chronic diseases. Saying no to these cultural foods or choosing healthier foods was viewed as a sign of defiance against social norms, and participants often felt that they could not decline when offered these foods or prepare them in a healthier way. As illustrated by the following excerpts:

*I realized that’s how [my grandmother] grew up. So, it’s a generational pattern and culture of eating certain foods at certain times and it has to be done that specific way. Because if the food is not made that way, it does not taste right, and then everyone will criticize you. (Participant #2, FG #4).*

*Then with my father’s Spanish heritage, when you visit someone’s house, you don’t go just to visit. They are going to feed you; you can’t say ‘no, I’ll just take a drink’. They are going to feed you, no matter what. (Participant #3, FG #4).*

#### Sub-theme 2: cultural views of stress and stress management vary across generations

Beyond the everyday life stressors such as routine family responsibilities, participants mentioned that they felt pressured by family members to work longer and harder, or otherwise, they would be deemed ‘lazy’. Furthermore, participants shared that stress was a taboo topic often viewed as inappropriate for discussion in their culture. One participant stated, *“With mental illness, it’s mainly, oh, nothing’s wrong. You’re just overreacting.*”

Many participants felt that stress and mental health problems were viewed as signs of weakness based on their culture, and those suffering from these problems were not expected to share them with family members. Despite these views on stress, participants still felt that stress often affects their lives and for some, it influences their everyday healthy lifestyle practices. For instance, some participants shared that stress made it difficult for them to eat healthfully, exercise, or sleep.

*I think as a culture, we work a lot, and we don’t have time to exercise, to really go out there and exercise like you need to, because in our culture, speaking for self, it’s a grind. (Participant #5, FG 3).*

*“I feel that stress from the COVID pandemic is what made everything worse with gaining weight and I haven’t really been eating right, sleeping right, or even exercising, which I really love to do.” (Participant #4, FG #4).*

There were differing generational approaches to stress management. Older participants dealt with stress by internalizing it, and their coping mechanisms for stress primarily involved religious activities such as prayer, gospel music, and church.

*But I do it healthy and I start my walking and start listening to my gospel music on my headset, and it’s just uplifting. Cause once you get that day started, you say that prayer, scatter your enemies and then it’s a good day. (Participant #2, FG #4).*

Conversely, younger participants were more likely to discuss their feelings with others and described using different coping strategies, including meditation and journaling. As one participant shared, “*The way I try to deal with my stress is meditating or writing my thoughts down in a journal.*”

### Theme 3: participants desire a more individualized approach to nutrition and physical activity interventions

#### Sub-theme 1: individualized nutrition and practical skills

Regarding nutrition education within health programs, participants wanted to learn very specific and individualized nutrition recommendations. For example, one participant shared, “*People don’t tend to know what fats, lipids, carbohydrates, and proteins are compatible with your body, so I think that would be meaningful*.” Many participants shared that their motivation for individualized nutrition recommendations stemmed from feeling overwhelmed by all the nutrition information they found, and nutrition information often conflicted between sources. As one excerpt illustrates, “*When you talk about different diets, there are 8 million different diets out there, but not every diet works for every person*.”

In addition, participants described components of the SHHC that were relevant and acceptable to them and that they would want to focus on. They described wanting to participate in a program that provided a variety of practical nutrition skills such as portion control, shopping on a budget, and cooking healthy recipes. As one participant stated about portion control, “*I’d go with a class that focuses on choosing healthier foods and portion sizes.*” Regarding shopping on a budget, a different participant shared, “*I’d like healthy food shopping on a budget. This is a high priority for me because sometimes it’s just so hard to find healthy food that’s not expensive*.” Concerning cooking healthy recipes, one participant stated, “*Provide a cooking class, and I’m there*.”

#### Sub-theme 2: accessible, enjoyable, and familiar physical activity

Almost all participants were interested in engaging in routine physical activity to get stronger and have more energy. Among the different suggested forms of physical activity, participants indicated the most interest in programs that were physically and economically accessible, such as walking and strength training. As these participants shared, “*I feel like anyone, minus disabled people, can do walking. It’s not something that’s hard to do like running. You can do it anytime, anywhere, and it can be done in a group*,” and “*I really enjoy lifting weights and strength training, so I would do that by myself or in a group*.”

Participants expressed hesitancy toward activities that were novel or different to them, such as dance cardio or Zumba, with some expressing that they would be more inclined to try if they were in a group setting. For example, one participant shared, “*I like doing things with other people. So, the walking group and Zumba, which I really like. I ranked high just for the group aspect*.”

#### Sub-theme 3: social support based on shared health goals

For most participants, family members with similar health goals played an important role in supporting their health behaviors. For instance, many participants mentioned specific family members directly or indirectly supporting them by going on walks together or helping them choose healthy food options. This was especially the case where the family members shared similar health goals, and where it was easier to support each other. For example, one participant said, “*My husband really monitors and watches what he eats, and that’s a good influence on me*.”

In instances where family members did not have the same common health goals, then participants were hesitant to include them in their health program. For example, one participant shared the pushback they received for weight training, “*I tend to get negative feedback from my family around me when lifting weights. It’s just like, oh, you shouldn’t be doing that. Aren’t you happy with who you are?*” For some, trying to achieve their health behavior goals was already difficult. They did not want the added burden of involving their family members or the responsibility of advocating for health behavior change within their households. For example, a participant shared, “*When I was in the gym, I was the only one going. So, if it was a family plan, it’s like, why do I have it? Nobody else goes*.” Finally, participants were more likely to engage in a health program that encouraged support from other group members. However, several participants specified that they preferred small groups or one-on-one rather than larger groups. Exemplary excerpts illustrating the desire for support include:

*… somebody had initiated that we start walking in the mornings. We did that for probably three or four months until it got super-hot. But that was a good support system. You just have somebody to walk with you every morning*. *(Participant #1, FG #2).*

*The buddy system for sure, because I remember when a few years ago me and my best friend started weight training. We always met up at the gym. That helped a whole lot: a buddy system and being held accountable.” (Participant #3, FG #2).*

## Discussion

This qualitative study aimed to understand the health goals and needs of African American/Black and Hispanic/Latinx women in urban communities and to identify how the SHHC intervention could be adapted to address their health goals related to CVD prevention. Our findings revealed awareness and a positive attitude toward the importance of a healthy lifestyle. However, there were many barriers to trying to practice healthier behaviors. There was a strong link between culture, food, and community. Our participants reported that some of their cultural-related communal food practices promote unhealthy eating behaviors. Additionally, discussions on stress were viewed as culturally inappropriate, with generational differences in stress relief activities. For future health programs, there was a desire for individualized nutrition while building various nutrition-related skills and accessible, enjoyable physical activities since family social support was only possible where there were shared health goals. There was a preference for programs offering small-group or one-on-one support opportunities to achieve health goals.

Prior health studies have shown that Americans have relatively good knowledge and positive attitudes toward the importance of healthy eating and exercise in promoting good health [14]. However, knowledge and positive attitudes alone do not translate to behavior [15]. According to previous research, while health knowledge is important, broader social, economic and environmental factors influence the capacity to adopt and sustain healthy behaviors [16]. Similarly, our results indicate that participants had good knowledge and positive attitudes regarding healthy lifestyle behaviors. Nevertheless, many participants faced obstacles to adopting or sustaining these behaviors, such as cost, time, and major life events, potentially exacerbated by social, economic and environmental constraints. Therefore, in addition to increasing awareness, health programs may address barriers to support the desired outcomes of healthier lifestyle behaviors. Furthermore, recognizing the influence of broader systemic influences, including social, economic, and environmental aspects, future studies should investigate these upstream factors in more depth to further understand their influence in impacting individual capacity to engage in a healthy lifestyle.

Participants in our study expressed a desire for individualized nutrition advice. Based on previous studies, this approach is likely to be more effective than generalized nutrition advice. Many dietary programs and guidelines often include a “one size fits all” nutrition approach but research evidence suggests that programs tailored to meet individual needs may be more acceptable and effective. For instance, the Food4Me trial, showed that adults who received individualized nutrition intervention had more significant changes in eating behavior after six months compared to the control who received conventional nutritional advice [17]. These findings suggest that programs that offer opportunities to tailor nutrition education to individual needs are more effective. Therefore, future programs, including the adaptation of SHHC, may be more effective by delivering tailored nutrition interventions highly personalized to individual health needs, motivations, and assessments.

Additionally, our findings indicate a desire for programs that offer accessible, enjoyable, and familiar forms of physical activity over novel ones. Although novelty can initially attract individulas to physical activity [18, 19], sustained engagement often relies on familiarity and enjoyment. For the women in this study, being familiar with the exercises in a health program, as well as enjoying and having access to the exercises, was preferred for engaging in and maintaining physical activity behavior. Undoubtedly, people often engage in novel physical activity where they are curious about the activities and their ability to perform them. However, this is usually short-lasting as the novelty period wears off, and many individuals discontinue [18, 20]. Therefore, what may cause positive, sustainable changes in physical activity behavior may be rooted in how strongly the activity is supported by the individual’s social and environmental context [20]. Thus, when designing interventions for African American/Black and Hispanic/Latinx urban women, activities such as walking and strength training (with or without weights) that are either physically and economically accessible would be more appealing and more likely to have continuity post-intervention—further understanding the barriers faced and how agency may be fostered through environmental and social mechanisms to encourage these women to engage in physical activity. These mechanisms may be as simple as having accessible transportation that is free or affordable to attend a healthy lifestyle program and creating an environment with support from fitness professionals, coaches, and peers, all of which can empower women to make positive physical activity choices.

While common barriers to engaging in healthy behaviors, such as weather, cost and time constraints, were identified in this study, it is interesting to note that major life events and transition times, such as going back to school, starting a family, and having a physical injury, were often the reasons why they stopped engaging in healthy behaviors. Life events and transitions often affect how people engage in healthy eating, physical activity and sleep, negatively impacting heart health [21, 22]. The American Heart Association recommends practical strategies to support people in maintaining healthier lifestyles during major life events and transitions by building resiliency [21]. Health programs such as the SHHC may be designed to increase awareness of the importance of maintaining a healthy lifestyle during major life events and transitions and offer relevant strategies applicable to such times.

In many cultures, including African American/Black and Hispanic/Latinx cultures, food has a social and communal role that is deeply entrenched as a channel of traditions, love, identity and acceptance [10, 23]. Consequently, culture influences the selection and preparation of food and even the frequency of consumption of certain foods [24]. For our study, while the culture around food positively fostered love and community, culture also contributed to unhealthy eating, including the consumption of large portions and increased consumption of energy-dense foods with high fat and added sugar. Culture also contributed to the social undermining of those who attempted to practice healthier behaviors. Our findings align with several qualitative studies that show a strong cultural tie to food in similar populations and the associated intake of foods high in calories, fats and sugars that can increase the risk of chronic diseases [23, 25]. Others have also found that social expectations to conform to cultural norms often hinder healthy behaviors [26]. Since food and culture are highly interconnected, future interventions could aim to preserve positive food culture while encouraging strategies for individuals to manage social norms and conflicts related to food and eating [27].

Additional findings related to culture included the challenge of discussing stress, as it was often viewed as a sign of weakness and a personal matter to be kept private. There were also generational differences in approaches to stress management. These findings are not unlike those of other studies that have shown similar cultural and generational views of stress and stress management [28, 29]. Individuals from the “baby boomer” generation often read or pray to manage stress [29], while younger generations (e.g., Gen Z) are more open to discussing stress and mental health [29]. Furthermore, the American Psychological Association found that millennials were more likely than older generations to engage in sedentary activities and other unhealthy behaviors such as alcohol abuse and smoking to relieve stress [28]. Considering the cultural and generational views of stress and stress management, health programs could consider fostering an environment of open and non-judgmental dialogue and communication to help break down the cultural barriers associated with discussing such issues. Additionally, they could consider promoting different healthy coping strategies relevant to the age of individuals in the intervention.

Social support has repeatedly been linked to better long-term health outcomes [30]. Individuals with strong social support are more likely to care for their physical and mental health than those with weaker social support [31]. In our study, when family members had shared health interests, they directly or indirectly encouraged healthier lifestyle behaviors for the participants. In situations where families did not share similar health interests, there was either social undermining or no support. These findings concur with those of a study exploring how social relationships influence the health behaviors of adults at risk of chronic diseases, where the attitudes and actions of family members are either facilitators for supporting or sabotaging health behaviors [26]. Their study also found that making healthier dietary changes was easier if spouses and family members were supportive, and that positive role modeling and encouragement from family members facilitated the adoption and maintenance of physical activity behaviors. In contrast, the lack of familial support affected motivation toward positive health behaviors [26].

Furthermore, in our present study, there was hesitancy to involve unsupportive family members in making health behavior changes. Many previous health programs have focused on leveraging family and friends’ social support to improve behavioral health [31]. However, with evidence that social undermining can be detrimental to practicing healthy behaviors [26], future health interventions should consider providing avenues to build social support from like-minded participants or family members with shared health goals, as well as building on self-determination to motivate and sustain behavior change in the absence of adequate social support [32].

A small sample size may be a limitation of our study, yet, it is important to note that thematic saturation was achieved, ascertained during the analysis process and supported by previous studies that indicate that more than 80–90% of all themes are discoverable using three to six FGs [33]. Most of the participants were African American/Black, so the findings may not entirely represent the experiences of Hispanic/Latinx women. We did identify commonalities across cultures, for instance, the role of food in culture and the cross-cultural views on stress. Our analysis, however, did not identify differences between African American/Black and Hispanic/Latinx participants. This is not to suggest that important differences may not exist. As such, future studies with larger samples of diverse women are needed to confirm or expand upon the study findings presented herein.

## Conclusion

The SHHC intervention and similar research health programs for Black/African American and Hispanic women in urban communities should emphasize individualized nutrition and practical skills for healthy eating with accessible, familiar, and enjoyable physical activities. Future interventions should also aim to resiliency to empower people with the skills to maintain positive health behaviors during major life events and transitions. In addition, stress management should cater to cultural and generational differences, while social support should be based on common health goals.

### Electronic supplementary material

Below is the link to the electronic supplementary material.

Supplementary Material 1

## Data Availability

Data described in the manuscript, code book, and analytic code will be made available from the corresponding author upon reasonable request.
