# Supplementary material for: A qualitative exploration of the health needs and goals of urban women to inform the tailoring and adaptation of Strong Hearts Healthy Communities: a community-based cardiovascular disease prevention intervention
Source: BMC Public Health. 2024 Jan 25;24:296. doi: 10.1186/s12889-024-17818-1 (PMC10811835; doi:10.1186/s12889-024-17818-1)
Supplement: Supplementary file 1 — Supplementary Material 1 [file 12889_2024_17818_MOESM1_ESM.pdf]

Strong Hearts Urban Communities Focus Group Guide

Welcome

Thank you all for being here and agreeing to complete the surveys & join the focus group. We appreciate your willingness to talk with us and to share your thoughts and ideas.

Introductions

My name is ___________________________ (interviewer) and I am with Texas A&M AgriLife's Institute for Advancing Health through Agriculture Healthy Living program. I will be facilitating our discussion. This is _____________________; she/he/they will be helping to facilitate and will be taking notes.

Purpose of Focus Groups

The Strong Hearts program was designed to help people learn about and practice things such as healthy eating and exercise. The original program was created for and tested in rural areas. It improved several important heart-related behaviors and risk factors, so we are modifying and testing it for urban settings.

That is the overall goal of our discussion with you today – to gain insight about how we should consider adjusting the program.

Ground Rules

1. WE WANT YOU TO DO THE TALKING.

   I'm going to be asking questions and follow up questions to guide the conversation, but we are interested in what you have to say, any experiences you have with previous programs, and ideas for what you would be interested in.

2. THERE ARE NO RIGHT OR WRONG ANSWERS

   Your experiences and opinions are important. We expect that you will have differing points of view, so please share yours. Please make sure that you respect what others are saying, even if you disagree with it.

3. CONFIDENTIALITY

   We want you to feel comfortable sharing when/if sensitive issues come up. So, please know that we will not share anything said in this discussion in a way that it can be linked back to you. We ask that you give individuals in this focus group the same courtesy.

4. WE WILL BE RECORDING THE DISCUSSION

   We want to capture everything you say and while _______________ does a great job at taking notes, she/he/they still may miss something. So, we would like to record our conversation so that we can use the recording to double check and make sure we have everything in our notes. To help with this, we ask that you please talk one at a time, speak in a voice as loud as mine, and avoid side conversations.

5. ADDITIONAL HOUSEKEEPING

   Our discussion will last 60 minutes. If you need to use the restroom during the discussion, feel free. They are located….

***Strong Hearts* Urban Communities Focus Group Guide**

As a reminder, we will not identify anyone by name in our report. If we use your words, we will simply say something like "one participant told us."

Do you have any questions of us before we begin?

Yes   If yes, answer questions

No   Okay, let's get started

For the first half of our discussion, we're going to ask some broad questions. We want to understand more about how you, as well as your friends and family, approach food, exercise, & stress. In the second half, we're going to ask more questions about the *Strong Hearts* program that are connected to the survey you just filled out.

1. To get started, please tell me your first name and how long you have lived in the Dallas area. You don't need to go in order. Feel free to just jump in.

2. We are interested in learning more about what you think you need to do to be healthier. On the survey, we asked you to choose 3 health goals; why did you choose the goals that you did?
    a. Do you have any health goals that were not on this list?
        i. Healthy eating goals?
        ii. Exercise goals?
        iii. Stress relief goals?
    b. What do you believe these goals would help you do?

3. Culture is defined as practices that are common in your community or the country you are from.
    a. How does culture influence the foods you eat?
    b. How does culture influence what exercises or physical activities you do?
    c. How does culture influence what you do to manage your stress?

4. Think about the scenarios that make it easier for you to be <u>healthy</u>. What impacts your healthy eating and exercise habits?
    a. If not addressed, probe about:
        i. Stress & emotions
        ii. Family
        iii. Friends
        iv. Community events
        v. Restaurants and fast food
        vi. Eating alone

   b. Who are the people in your life who may be cheerleaders and/or do new healthy things with you – like this program, walking at work, or trying a new recipe? *Note: Not interested in names, but in their role/relationship.*
   c. Who are the people who you would rely on for support when making health behavior changes?

5. Think about the scenarios that lead you to do things that are <u>unhealthy</u>. What are the things that lead you to unhealthy eating and being inactive?
   a. If not addressed, probe about:
      i. Stress & emotions
      ii. Family
      iii. Friends
      iv. Community events
      v. Restaurants and fast food
      vi. Eating alone
   b. Are there people in your life that you think would not support you trying to be healthier? Why do you think that?

6. So now that we've talked about things that influence healthy and unhealthy behaviors, if you were thinking about joining a program or group to improve your health, what would you want to learn or do?
   a. Why would that be important to you?
   b. What do you think would be helpful for a program to focus on to address things that lead to unhealthy eating, avoiding exercise, or stress?
   c. How could a program or group help support you with addressing this social support or impact?
      i. Sessions that practice handling different social scenarios?
      ii. Participate in the program with those people?
      iii. Invite those people to certain classes or special group events?

Strong Hearts is a 3-month program (12 weeks) with classes twice a week for 45-60 minutes. During each class, the group focuses on healthy eating and exercise with some stress relief activities.

7. In the survey, we asked you to identify which group fitness classes you would want to do, including aerobic dance, walking, and lifting weights.
   a. Why did you rate these the way you did?
   b. What are some other exercises you would want to try?
   c. What equipment, if any, do you have at home or work to exercise?
      i. If not addressed, probe about hand weights, yoga/fitness mats, etc.
   d. What resources do you have in your neighborhood for exercise?
      i. If not addressed, probe about gyms, tracks, trails, sidewalks, etc.

8. The Strong Hearts program has nutrition and healthy eating lessons, group discussions, and hands-on activities. On the survey, we asked you to rate how helpful you think some of the topics would be.
    a. Why did you rate these the way you did?
    b. Are there other topics you think would be helpful? Are there other topics you would rather learn about?

9. The Strong Hearts program has recipes for healthy snacks were brought to some classes for people to try. On the survey, we asked you to identify the ones you would want to try the most and the ones you would be less likely to try.
    a. How did you select the ones to try? How about the ones you didn't think you would want to try?
    b. What, if any, foods that are important to your family or culture that you are concerned aren't healthy?

10. The Strong Hearts program also tries to help address stress and mindfulness through guided activities, breathing techniques, and discussions. We asked you on the survey about which activities you've done before to manage your stress.
    a. Are there other activities that you do for stress relief?
    b. How often do you do stress relief activities?
    c. How do you feel about bringing stress relief activities into your life?
    d. What other activities would you want to do or learn to manage your stress?

11. The Strong Hearts program also helps you identify your personal goals related to health, with ongoing support from the group. We asked on the survey about some different types of social support from the group.
    a. Why did you choose to rate these how you did?
    b. Are there other ways to connect that would be helpful?

12. From our discussion today, what things would make you most likely to join Strong Hearts?
    a. What things would keep you from joining a group like Strong Hearts?
    b. Prompt for: compensation, online vs. in-person, location, joining with friends/family

13. What additional thoughts, if any, do you have about any topics today or something we may have missed?
